# Dynamic induction of the myelin‐associated growth inhibitor Nogo‐A in perilesional plasticity regions after human spinal cord injury

**DOI:** 10.1111/bpa.13098

**Published:** 2022-06-13

**Authors:** Carmen Schwaiger, Thomas Haider, Verena Endmayr, Tobias Zrzavy, Victoria E. Gruber, Gerda Ricken, Anika Simonovska, Simon Hametner, Jan M. Schwab, Romana Höftberger

**Affiliations:** ^1^ Division of Neuropathology and Neurochemistry, Department of Neurology Medical University of Vienna Vienna Austria; ^2^ Department of Orthopedics and Trauma Surgery Medical University of Vienna Vienna Austria; ^3^ Department of Neurology Medical University of Vienna Vienna Austria; ^4^ Department of Pediatrics and Adolescent Medicine Medical University of Vienna (Affiliated Partner of the ERN EpiCARE) Vienna Austria; ^5^ Center for Medical Physics and Biomedical Engineering Medical University of Vienna Vienna Austria; ^6^ The Belford Center for Spinal Cord Injury and Departments of Neurology, Physical Medicine and Rehabilitation and Neurosciences The Ohio State University Columbus Ohio USA

## Abstract

The myelin‐associated inhibitor Nogo‐A (Reticulon 4, RTN4) restricts axonal outgrowth, plasticity, and neural circuitry formation in experimental models of spinal cord injury (SCI) and is targeted in clinical interventions starting treatment within 4 weeks post‐SCI. Specifically, Nogo‐A expressed by oligodendroglia restricts compensatory neurite sprouting. To interrogate the hypothesis of an inducible, lesion reactive Nogo‐A expression over time, we analyzed the spatiotemporal Nogo‐A expression at the spinal lesion core (region of tissue necrosis and axonal damage/pruning) and perilesional rim (region of plasticity formation). Spinal cord specimens of SCI subjects (*n* = 22) were compared to neuropathologically unaltered controls (*n* = 9). Nogo‐A expression was investigated ranging from acute (0–3 days), early subacute (4–21 days), late subacute (22–90 days) to early chronic–chronic (91 days to 1.5 years after SCI) stages after SCI. Nogo‐A expression in controls is confined to motoneurons in the anterior horn and to oligodendrocytes in gray and white matter. After SCI, the number of Nogo‐A^+^ and TPPP/p25^+^ oligodendrocytes (i) inclined at the organizing perilesional rim specifically, (ii) increased further over time, and (iii) peaked at chronic stages after SCI. By contrast, at the lesion core, the number of Nogo‐A^+^ and TPPP/p25^+^ oligodendrocytes did not increase. Increasing numbers of Nogo‐A^+^ oligodendrocytes coincided with oligodendrogenesis corroborated by Nogo‐A coexpression of Ki67^+^, TPPP/p25^+^ proliferating oligodendrocytes. Nogo‐A oligodendrocyte expression emerges at perilesional (plasticity) regions over time and suggests an extended therapeutical window for anti‐Nogo‐A pathway targeting interventions beyond 4 weeks in patients after SCI.

## Graphical abstract

Nogo‐A oligodendrocytes expression emerges at perilesional regions over time and suggests an extended therapeutical window for anti‐Nogo‐A pathway trageting interventrions beyond four weeks in patients after spinal cord injury.

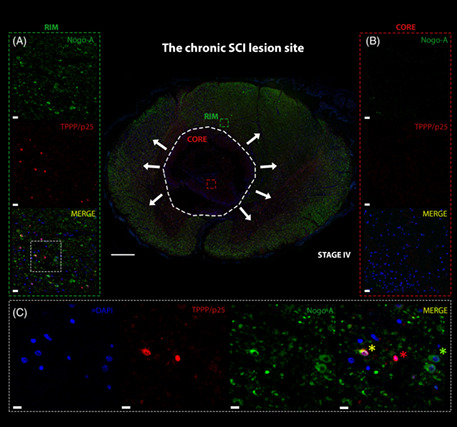

## 1 INTRODUCTION

Spinal cord injury (SCI) is a consequence of mechanical traction and/or compression to the spinal cord, affecting more than 27 million people worldwide [1]. Damage to the spinal cord leads to loss of sensory and/or motor function below the level of injury and results in life‐long disability in most cases. The functional recovery following SCI depends on various factors including the extent of the primary and secondary injury, progressive demyelination, and the level of injury [2, 3, 4, 5, 6]. Numerous studies in rodents demonstrated that axonal sprouting after trauma occurs spontaneously [7, 8, 9]. The myelin‐associated inhibitory factor (MAIF) Nogo‐A (Reticulon 4, RTN4), a transmembrane protein expressed by oligodendrocytes and motoneurons in the spinal cord inhibits axonal outgrowth and sprouting following experimental SCI [10, 11]. While the function of Nogo‐A expressed by neurons remains elusive, the inhibitory effect on axonal growth has been attributed to Nogo‐A synthesizing oligodendrocytes [12].

Nogo‐A restricts neuroaxonal plasticity via the Nogo‐receptor 1 (NgR1) and the paired immunoglobulin‐like receptor B (PirB), causing downward signaling by activating the Ras homolog family member A (RhoA) pathway leading to destabilization of the cytoskeleton and inhibition of axonal outgrowth [13, 14, 15, 16, 17]. Preclinical studies in rodent and primate models revealed that anti‐Nogo‐A treatment, using functional‐blocking antibodies improved axonal sprouting, neuroplasticity, and circuitry formation after SCI [18, 19, 20, 21, 22, 23]. Hence, blockade of the MAIF Nogo‐A is considered to be a plausible therapeutic strategy to enhance neuroaxonal plasticity and repair [18, 19, 20, 21, 24, 25]. The corresponding first in‐human trial with intrathecal application of anti‐Nogo‐A (ATI355 infusion) assessing feasibility and safety in 52 participants has been completed [26]. At present, biological anti‐Nogo‐A therapy has entered clinical Phase II trial testing (NCT03935321; https://clinicaltrials.gov/).

While rodent studies suggest an overall stable, nonreactive Nogo‐A expression after SCI, the concept of injury‐related dynamic myelin inhibition has been proposed earlier by Martin Raff's group [10]. Over the last two decades, this concept has been supported by accumulating substantial evidence demonstrating that oligodendrocytes, the main cellular source of MAIF, respond to SCI resulting in oligodendrogenesis and remyelination starting at 2 weeks after SCI and lasting through chronic SCI conditions [2, 27, 28, 29, 30]. Substantial oligodendrogenesis after SCI suggests also a dynamic regulation of Nogo‐A synthesis after human SCI.

Even though neuroplasticity can occur along the entire neuraxis, plasticity responses confined to the lesion site and its adjacent particular perilesional areas have been of particular interest and considered relevant for propagating functional recovery [31, 32]. To date, investigations of Nogo‐A in human SCI were restricted to remote areas such as sites of Wallerian Degeneration while characterization of Nogo‐A expression in lesions and perilesional regions is still missing [33].

Given the attributed functional relevance of oligodendroglial Nogo‐A synthesis in inhibiting neuroaxonal plasticity, the aim of this study was to determine whether oligodendroglial Nogo‐A expression is modulated after human SCI [12]. Specifically, we assessed oligodendroglial Nogo‐A expression in relation to acute axonal injury/disturbed axonal transport and in regions of axonal damage/pruning (core) and regions of plasticity formation (perilesional rim) [34, 35].

## 2 MATERIALS AND METHODS

### 2.1 Human post‐mortem spinal cord tissue

**FIGURE 1:**
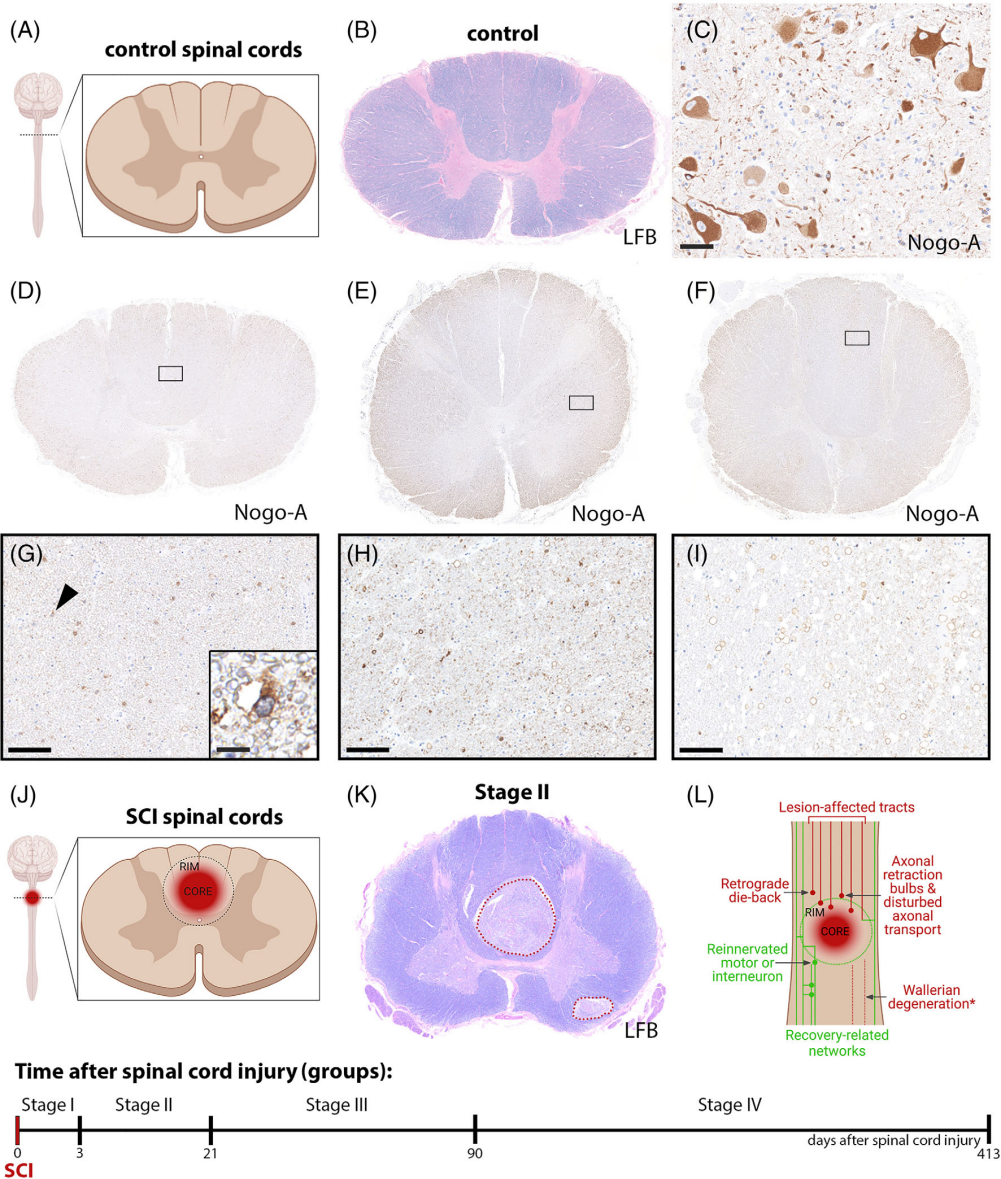
Characterization of areas of interest in human postmortem spinal cord sections. (A) Schematic illustration of sectioning of control spinal cords. (B) Representative control spinal cord section stained with Luxol fast blue‐periodic acid‐Schiff (LFB). (C) Immunohistochemical detection of Nogo‐A^+^ motoneurons in the anterior horn of the human spinal cord (scale bar: 10 μm). (D–I) Nogo‐A^+^ oligodendrocytes in representative control spinal cords without neurological deficits. Further, the black arrow marks a Nogo‐A^+^ oligodendrocyte (amplified insert; G–I; scale bar: 100 μm; inset in G scale bar: 10 μm). SCI, spinal cord injury. (J) Schematic illustration of the defined regions of interest “lesion core” and “lesion rim” in an injured spinal cord. (K) Representative image of an LFB stained injured spinal cord section (stage II; scale bar = 1 mm). (L) Simplified lesion panorama illustrating different areas and mechanisms with reference to lesion topology and tissue reorganization. Nogo‐A expression was differentially assessed at regions of prevailing axonal damage/pruning (spinal lesion core) and plasticity formation (perilesional rim) after human SCI. Neurobiological processes were ascribed to those regions relevant to post‐injury circuity formation. Disturbed axonal transport is a feature of axonal injury and can, after full deafferentation, give rise to axonal spheroids/retraction bulbs. Injured axons are exposed to a growth inhibitory milieu to which Nogo‐A contributes. Degeneration of axons can occur in retrograde (die‐back) or anterograde direction (Wallerian degeneration). While Nogo‐A expression has been examined in more lesion remote areas of axonal degeneration (Wallerian degeneration) after human SCI earlier [33, 37] information on Nogo‐A expression at the lesion core and rim is lacking. Lesion‐affected axonal tracts are depicted in red and elements of the recovery‐related networks (plasticity) in green. (A) and (J) were created with Biorender.com

This study was performed on paraffin‐embedded archival autopsy material using a collection of spinal cord autopsy tissues derived from 22 human cases of traumatic SCI and 9 controls with neuropathologically unaffected spinal cords as reported earlier [36]. All specimens were collected during the past decades (between 1960 and 2018) in the archive of the Division of Neuropathology and Neurochemistry, Department of Neurology, Medical University of Vienna. All available medical records, documents, and autopsy data were examined. Nine control cases without neuropathological changes in the spinal cord were included (Figure 1A—Schematic illustration; Figure 1B—Luxol Fast blue/periodic acid‐Schiff stainings [LFB]). Nogo‐A expression in controls was quantified in representative areas of white matter (Figure 1D–I). Patient demographics from SCI and controls are summarized in Table 1. Regions of interest included the lesion core (core) equally to the definition “Zone 1” as well as the directly surrounding tissue margin (rim) to definition of “Zone 2” as previously defined by Fleming et al. (Figure 1J) [36, 38]. We used hematoxylin and eosin and LFB stainings to characterize demyelinated/injured areas (Figure 1K). A detailed neuropathological and inflammatory characterization has been provided recently [36]. Traumatic SCI cases were divided into four groups according to the respective stage after SCI: stage I: acute (0–3 days), stage II: early subacute (4–21 days), stage III: late subacute (22–90 after SCI), and stage IV: early chronic – chronic (91 days to 1.5 years) [36]. This study was approved by the ethics committee of the Medical University of Vienna (EK nos.: 1451/2018 and 1636/2019). Anonymized data not published in this article will be made available upon request from any qualified investigator.

**TABLE 1:** Clinical demographics of patients with traumatic spinal cord injury and controls with neuropathically unaffected spinal cord

Case ID	Sex	Age at death (years)	Spinal cord level of injury/interest	Spinal cord injury cause	Days post spinal cord injury (survival)	Stage	Cause of death	Type of spinal cord injury
SCI# 1	Male	33	Thoracic	Gunshot	<1	I	Cardiovascular failure	Massive compression
SCI# 2	Male	79	Cervical	Fall	1	I	Cardiovascular failure	Contusion
SCI# 3	Male	30	Cervical	Fall	2	I	Respiratory insufficiency	Contusion
SCI# 4	Female	79	Cervical	Fall	2	I	Pulmonary embolism	Massive compression
SCI# 5	Male	53	Cervical	fall	4	II	Cardiovascular failure	Contusion
SCI# 6	Female	75	Cervical	Fall	4	II	Cardiovascular failure	Contusion
SCI# 7	Male	50	Cervical	Fall	5	II	Pulmonary embolism	Contusion
SCI# 8	Female	35	Thoracic	Massive traumatic prolapsed disc	13	II	Pulmonary embolism	Massive compression
SCI# 9	Male	45	Cervical	Fall	14	II	Unknown	Contusion
SCI# 10	Male	37	Cervical	Fall	15	II	Cardiovascular failure	Contusion
SCI# 11	Male	71	Cervical	Fall	18	II	Cardiovascular failure	Contusion
SCI# 12	Male	19	Lumbal	Car accident	18	II	Renal failure	Contusion
SCI# 13	Male	15	Cervical	Fall	19	II	Unknown	Contusion
SCI# 14	Male	39	Cervical	Fall	25	III	Respiratory insufficiency	Massive compression
SCI# 15	Male	22	Cervical	Fall	36	III	Respiratory insufficiency	Contusion
SCI# 16	Male	80	Cervical	Fall	48	III	Pulmonary edema	Contusion
SCI# 17	Male	65	Cervical	Fall	89	III	Hepatorenal failure	Contusion
SCI# 18	Male	61	Cervical	Fall	91	IV	Pulmonary embolism	Contusion/cyst
SCI# 19	Female	34	Thoracic	Car accident	210	IV	Pulmonary edema	Contusion/cyst
SCI# 20	Female	24	Cervical	Car accident	352	IV	Cardiovascular failure	Contusion/cyst
SCI# 21	Male	56	Cervical	Massive traumatic prolapsed disc	365	IV	Cardiovascular failure	Contusion/cyst
SCI# 22	Male	58	Cervical	Fall	413	IV	Cardiovascular failure	Contusion/cyst
CO# 1	Female	70	Cervical	–	–	–	Pneumonia	–
CO# 2	Male	32	Cervical	–	–	–	Hepatic cirrhosis	–
CO# 3	Male	73	Cervical	–	–	–	Multiple organ failure	–
CO# 4	Male	40	Cervical	–	–	–	Cardiovascular failure	–
CO# 5	Female	53	Cervical	–	–	–	Cardiovascular failure	–
CO# 6	Female	71	Cervical	–	–	–	Pneumonia	–
CO# 7	Male	73	Cervical	–	–	–	Cardiovascular failure	–
CO# 8	Female	32	Cervical	–	–	–	Sepsis	–
CO# 9	Male	56	Thoracic	–	–	–	Aspiration and asphyxia	–

*Note*: SCI, spinal cord injury; CO, control cases; stage I: acute (0–3 days), stage II: early subacute (4–21 days), stage III: late subacute (22–90 days), and stage IV: early chronic–chronic (91 days to 1.5 years) stages after spinal cord injury.

### 2.2 Immunohistochemistry

Immunohistochemistry (IHC) was performed on formalin‐fixed paraffin‐embedded human spinal cord sections. After deparaffinization and rehydration, sections were incubated in 3% hydrogen peroxidase in methanol to block endogenous peroxidase activity. The following primary antibodies were used: anti‐Nogo‐A (specificity according to the manufacturer: recognizing a 175 amino acid fragment of the first cytoplasmic domain; *not* recognizing Nogo‐B or Nogo‐C; 1:1000; rabbit, polyclonal, Millipore, AB5888; pH 6 citrate buffer), anti‐TPPP/p25, which has been shown as reliable and specific marker for mature, myelinating gray and white matter oligodendrocytes either in unaltered controls or diseased multiple sclerosis lesions [39, 40, 41] (1:1000; pH 6 citrate buffer) and anti‐APP (amyloid precursor protein; 1:8000, Chemicon®, pH 6 citrate buffer) to identify disturbed axonal transport as a feature of axonal injury. Sections were incubated with the primary antibody (dilutions as described above) in antibody diluent/blocking solution (DAKO®) overnight at 4°C. Samples were washed three times with TRIS/HCl buffer, followed by 25 min incubation at room temperature (RT) with EnVision® Flex+ kit as detection system (DAKO®) and 3′,3‐diaminobenobenzidine (DAB) as chromogen was used for 10 min at RT to visualize antibody reaction.

For double labeling using the rabbit polyclonal primary antibodies, Nogo‐A (1:1000) and mouse monoclonal primary antibody and proliferation marker Ki‐67 (1:200, MIB‐1, DAKO®), the same antigen retrieval technique (pH 9 EDTA buffer) was used. Visualization of Nogo‐A was performed by the use of alkaline phosphatase‐conjugated secondary antibody for subsequent development with Fast Blue BB salt (4‐Benzoylamino‐2,5‐diethoxybenzene‐diazonium chloride hemi salt; blue; Sigma‐Aldrich®). Visualization of Ki‐67 was conducted with the use of a biotinylated secondary antibody and a horseradish peroxidase‐conjugated streptavidin reagent for subsequent development with amino ethyl carbazole (AEC; red; DAKO®). Slides were evaluated using a light microscope.

### 2.3 Immunofluorescence

Double immunofluorescence labeling was performed on deparaffinized spinal cord sections and blocked with 3% hydrogen peroxidase in methanol. For antigen retrieval, all slides were heated at pH 6 in citrate buffer. To reduce auto‐fluorescence of aldehydes, all slides were incubated in 1% NaBH_4_ (Merck GmbH, 106371) in TRIS/HCl buffer for 2 min at RT. Primary antibodies were used as follows: Nogo‐A 1:500 and TPPP/p25 1:500, pH 6 citrate buffer, overnight 4°C; NG2 (Millipore, rabbit, polyclonal) 1:200 and Ki67 1:200, pH 9 EDTA buffer, overnight 4°C. The following secondary antibodies were used for 30 min at RT in the dark: goat anti‐rabbit AF488 (Alexa Flour® 488, Jackson ImmunoResearch Laboratories) and donkey anti‐mouse Cy3™ (Jackson ImmunoResearch Laboratories). Cell nuclei were stained with DAPI (4′,6‐Diamidin‐2‐phenylindol), autofluorescence of lipophilic structures was blocked with 1% Sudan black for 3 min at RT and sections were mounted with Vectashield (H‐1200, Vector Laboratories). For negative controls, the primary antibodies were omitted (data not shown). Antibody binding was visualized by fluorescence microscopy performed with an OLYMPUS BX63 fluorescence microscope and Olympus cellSens software and/or the Vectra Polaris Multispectral Imaging and Whole Slide Scanning System (PerkinElmer).

### 2.4 Quantitative and qualitative assessment

Anti‐Nogo‐A, anti‐TPPP/p25, and anti‐APP stained IHC slides were digitalized using a NanoZoomer (Hamamatsu Photonics K.K.). The corresponding software NPD.Viewer2 was used to export the regions of interest (lesion core vs. lesion rim) of an area of 0.4263 mm^2^ (High Power Field). We used morphological characteristics and double staining with the oligodendrocyte marker TPPP/p25 to differentiate the expression of Nogo‐A on oligodendrocytes and neurons [10, 11]. Quantification of Nogo‐A^+^ oligodendrocytes, TPPP/p25, and APP was then performed digitally and expressed as cell counts per area. Co‐staining/double labeling of Nogo‐A + TPPP/p25 and NG2 + Ki67 were further performed and evaluated in a descriptive manner on representative sections.

### 2.5 Statistical analysis

Data were analyzed using SPSS®Statistics (IBM®, Version 27). For comparison of TPPP/p25 and Nogo‐A expression between the different cohorts, we utilized the Kruskal–Wallis test for multiple group comparison and the Mann–Whitney *U*‐test for comparison between two cohorts. The Wilcoxon signed‐rank test was used to compare Nogo‐A expression between lesion core and rim. We calculated the two‐tailed Pearson's correlation coefficient for correlation analysis of TPPP/p25, Nogo‐A, and time after SCI. A *p* value < 0.05 was considered statistically significant.

### 2.6 Data availability

All data can be made available from the corresponding author upon reasonable request and after approval from the ethics review board at the Medical University of Vienna, Vienna, Austria.

## 3 RESULTS

### 3.1 Demographic data

Spinal cord specimens from 22 traumatic SCI cases and nine neuropathologically unaffected controls were assessed (Table 1). Mean age of the SCI patients was 48.1 years (range 15–80 years). The sex ratio was 73%:27% (male:female). The cohort mainly comprised tetraparetic patients with cervical SCI level (81.8%). Paraparetic patients (18.2%) composed of thoracic lesions in three individuals (13.6%), and in one case (4.5%) the lumbar spinal cord was affected. Distribution of injury level was in line with recently published SCI demographics [42]. Underlying etiology for SCI was heterogeneous. Also in line with epidemiological data, the most frequent causes of injury included falls, followed by traffic accidents and mass disc herniations (Table 1) [1]. In one case a gunshot injury had caused the spinal lesion and resulted in early fatal outcome (<24 h). The cause of death after SCI ranged from cardiovascular failure to respiratory insufficiency and pulmonary embolism (Table 1). Nine controls with a comparable mean age of 55.6 years (range 32–73 years) and a comparable sex distribution of six males and three females (66%:33%), were included (Table 1).

### 3.2 Nogo‐A expression in the neuropathologically unaltered control spinal cord

In the gray matter of the spinal cord, Nogo‐A was expressed to a variable extent by motoneurons, including their axons and dendrites, in the anterior horn, in the nucleus dorsalis (Clarke's neurons), as well as by interneurons and marginal zone neurons of the posterior horn (Figure 1C). In the white matter, extensive Nogo‐A expression was observed in oligodendrocytes and to a lesser extent in structural myelin. The subcellular Nogo‐A expression pattern was mainly confined to the cytoplasm. The detected expression pattern in control spinal cord tissue matches earlier descriptions reported independently (Figure 1D–I) [43].

### 3.3 Nogo‐A and TPPP/p25 expression by oligodendrocytes after SCI—A spatial analysis

**FIGURE 2:**
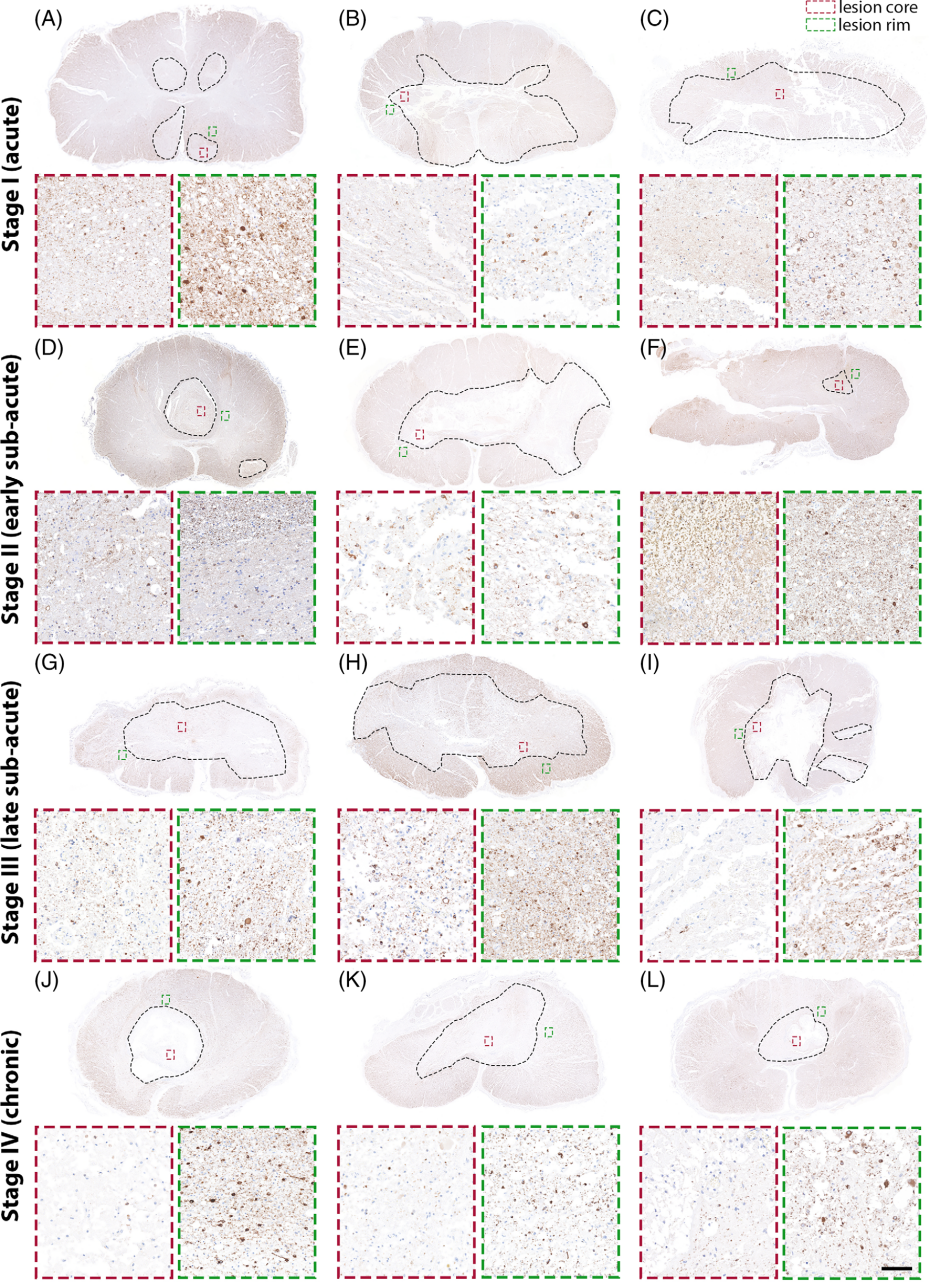
Differential Nogo‐A expression after spinal cord injury over time from stages I to IV: Upregulation at the peri‐lesional rim and reduction at the necrotic lesion core. Black dashed edgings mark the lesion core, red square = core area, green square = lesion rim. (A–C) stage I (acute), days 0–3 after SCI. (D–F) stage II (early subacute), days 4–21. (G–I) stage III (late subacute), days 22–90. (J–L) stage IV (chronic), days 91–413 days post‐SCI (scale bar: 50 μm). SCI, spinal cord injury.

**FIGURE 3:**
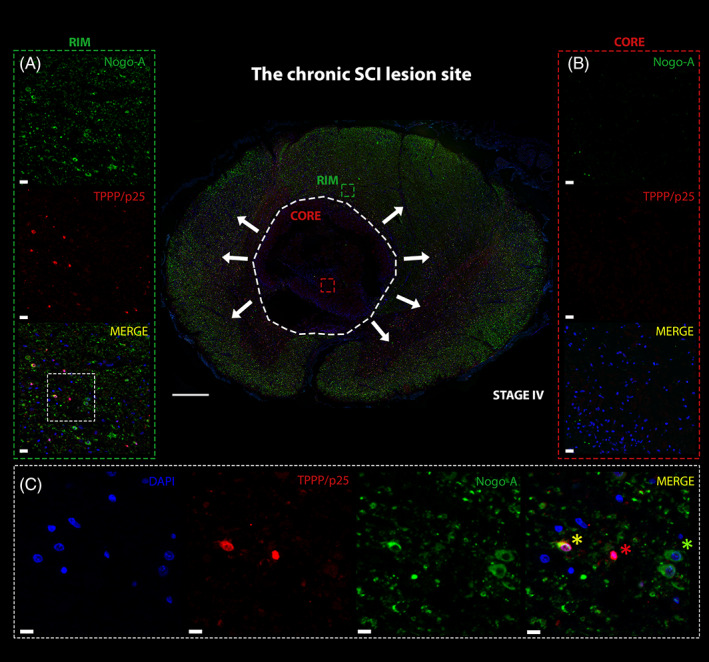
Nogo‐A is expressed by a subpopulation of TPPP/p25^+^ oligodendrocytes after SCI. Representative Nogo‐A and TPPP/p25 expression at the chronic SCI lesion (210 days after SCI) illustrating Nogo‐A and TPPP/p25 coexpression by oligodendrocytes. Lesion core is demarcated by a white dashed line (spinal cord overview; scale bar: 800 μm). (A) Peri‐lesional rim (green square, left) and (B) core (red square, right) with Nogo‐A^+^, TPPP/p25^+^, and double‐positive oligodendrocytes. While the lesion core has become devoid of Nogo‐A^+^ cells, the peri‐lesional rim demonstrates abundant Nogo‐A expression forming a perpendicular gradient with increased expression of the outer rim (white arrows). Counterstained nuclei indicated by DAPI (blue; scale bar: 50 μm). (C) 40× magnification of the peri‐lesional rim demonstrates Nogo‐A^+^ (green asterisk), TPPP/p25^+^ (red asterisk), and double‐positive oligodendrocytes (yellow asterisk; scale bar: 10 μm). SCI, spinal cord injury.

**FIGURE 4:**
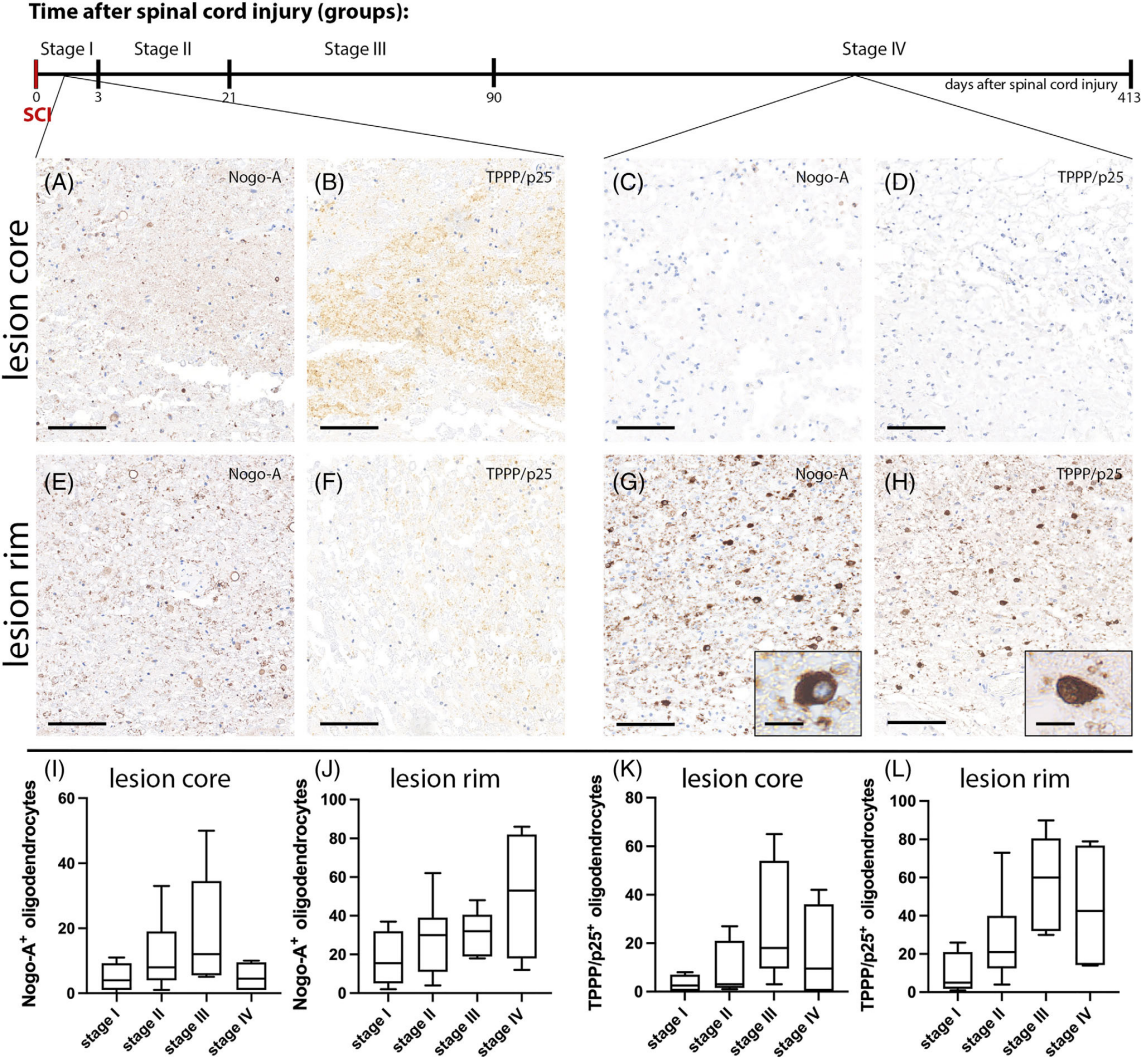
Nogo‐A and TPPP/p25 expression in oligodendrocytes within the lesion core and peri‐lesional rim after spinal cord injury (SCI) throughout stages I–IV. (A, B, E, F) Representative immunostainings against Nogo‐A and TPPP/p25 in stage I (2 days after SCI) and (C, D, G, H) stage IV (210 days after SCI; A, B, C, D) in the lesion core and (E, F, G, H) lesion rim (scale bar: 50 μm, scale bar insets: 10 μm). (I–L) Boxplots of quantified Nogo‐A^+^ and TPPP/p25^+^ oligodendrocytes illustrating the four respective stages after SCI (stage I: acute, 0–3 days; stage II: early subacute, 4–21 days; stage III: late subacute, 22–90 days; stage IV: early chronic–chronic, 91 days to 1.5 years after SCI) in (I) lesion core and (J) lesion rim stages I–IV Nogo‐A^+^ oligodendrocytes. (K) TPPP/p25+ oligodendrocytes in the lesion core. (L) Boxplots of TPPP/p25^+^ oligodendrocytes in the lesion rim/0.4263 mm2, with a significant difference of TPPP/p25 expression among stages (*p* < 0.05).

Different areas with regards to the lesion topology were compared as illustrated in Figure 1J,K. Nogo‐A expression was differentially assessed at regions of prevailing axonal damage/pruning (spinal lesion core) and plasticity formation (perilesional rim) after human SCI. A number of neurobiologically relevant processes have been ascribed to those regions relevant to post‐injury circuitry formation (Figure 1L). Nogo‐A positive oligodendrocytes were examined in representative lesion core and lesion rim areas in stages I–IV (Figure 2). Oligodendrocytes were examined based on morphological criteria and expression of TPPP/p25. Multicolor double‐labeling immunofluorescence was applied to verify the cellular source of Nogo‐A expression in oligodendrocytes. Nogo‐A and TPPP/p25 coexpression were detected in spinal cords throughout all four stages at variable degrees in lesion core and rim (Figure 3A–C). Nogo‐A expression was neither restricted to TPPP/p25^+^ cells nor vice versa (Figure 3C). Dynamic fluctuations of Nogo‐A and TPPP/p25 expression patterns were found in both the lesion core and lesion rim, respectively (Figure 4A–L). An intense, robust Nogo‐A expression by surviving oligodendrocytes was detected after SCI (Figure 4G).

#### 3.3.1 Lesion rim

Overall, the number of Nogo‐A^+^ oligodendrocytes at the lesion rim was elevated and significantly higher compared to the lesion core (*p* < 0.001; Figure 4I,J). A positive correlation between TPPP/p25 and Nogo‐A expression was observed in the lesion rim (*r* = 0.656, *p* < 0.01; Figure 4J,L).

#### 3.3.2 Lesion core

Compared to controls, a significantly lower number of Nogo‐A^+^ oligodendrocytes was detected at the lesion core (*p* < 0.01; Figure 4A). To assess whether Nogo‐A expression is associated with disturbed axonal transport, a hallmark of axonal injury, we quantified ß‐APP‐positive axonal spheroids/retraction bulbs. As demonstrated earlier [36], ß‐APP‐positive axonal spheroids/retraction bulbs demarcate at the lesion border from day 4 onwards and subside with increasing time after SCI (*r* = −0.507, *p* < 0.05). However, number of Nogo‐A^+^ oligodendrocytes did not correlate with the number of APP^+^ axonal spheroids/retraction bulbs. Nogo‐A^+^ motoneurons were detected in a number of cases and were preserved dependent on the lesion size displaying a mild to moderate cytoplasmatic expression of Nogo‐A (Figure 1C).

### 3.4 Nogo‐A expression from acute to chronic SCI—A temporal analysis

**FIGURE 5:**
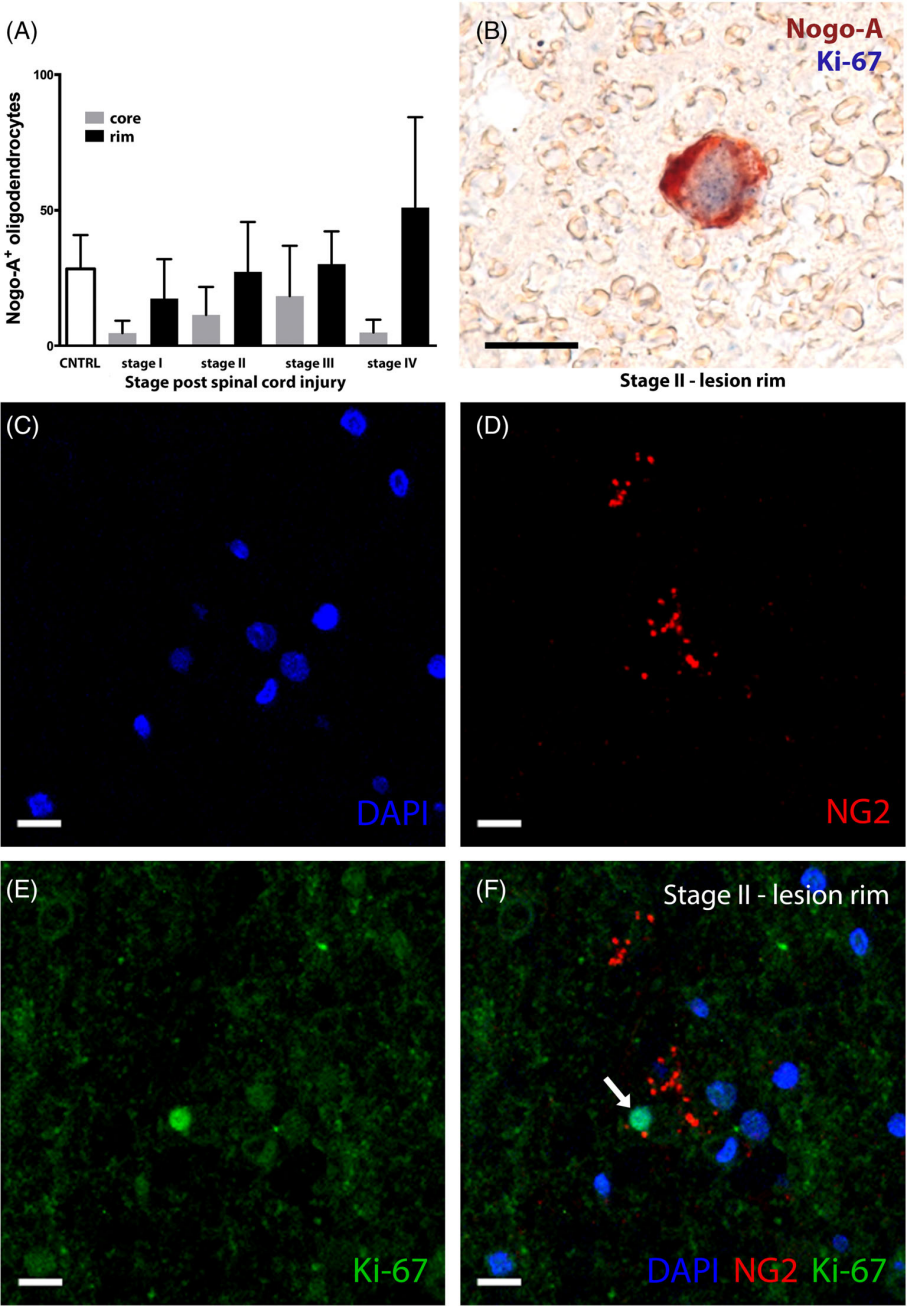
Dynamic Nogo‐A expression by oligodendrocytes from acute to chronic spinal cord injury coincides with oligodendrogenesis. (A) Time‐depending expression of Nogo‐A by oligodendrocytes in the lesion core and lesion rim after spinal cord injury, CNTRL = controls; stage I: acute, 0–3 days; stage II: early subacute, 4–21 days; stage III: late subacute, 22–90 days; stage IV: early chronic–chronic, 91 days to 1.5 years post‐spinal cord injury (SCI). (B) Double‐positive oligodendrocyte co‐expressing Nogo‐A^+^ (red) and proliferation marker Ki‐67^+^ (blue) as depicted within the lesion rim corroborate a role in post‐SCI oligodendrogenesis (stage II after spinal cord injury; scale bar: 10 μm). (C–F) Immunofluorescence co‐labeling of NG2^+^ and Ki67^+^ oligodendrocytes after early‐subacute spinal cord injury (stage II). Nuclear staining with DAPI, NG2 in red (cy3), Ki‐67 (MIB1, DAKO) in green (AF488). Double‐positive oligodendrocyte marked with white arrow (scale bars: 10 μm).

Temporal fluctuations of Nogo‐A^+^ oligodendrocytes over time after SCI were investigated (Figure 5A). To analyze time‐dependent expression dynamics, we grouped our cases into four stages based on time after SCI as defined previously [42].

#### 3.4.1 Lesion rim

A trend towards a correlation of Nogo‐A^+^ oligodendrocytes at the lesion rim with increasing time after SCI was found (*r* = 0.417, *p* = 0.053). Concordant with a lesion‐reactive oligodendrogenesis [28], the number of TPPP/p25^+^ oligodendrocytes at the lesion rim increased in a time‐dependent manner after SCI (*r* = 0.536, *p* < 0.05). A persistent increase of Nogo‐A^+^ cells was observed continuing until the chronic phase in the lesion rim (stage IV; Figure 4J) with a subpopulation demonstrating nuclear coexpression of the proliferation marker Ki‐67 (Figure 5B). We could also detect double‐labeled Ki‐67^+^ and NG2^+^ oligodendrocytes in the lesion rim in stage II after SCI (Figure 5C–F). With multiple group comparisons, we detected a statistically significant difference of TPPP/p25 expression within the lesion rim between all individual stages (*p* < 0.05; Figure 4L).

#### 3.4.2 Lesion core

A mild and only transient increase of Nogo‐A^+^ cells was detected in the lesion core up to stage III followed by a subsequent decline with entering chronic SCI (stage IV; Figure 3I). A comparable trend over time was observed for TPPP/p25^+^ oligodendrocytes (Figure 3K,L). Compared to the lesion rim, only a modest increase of Nogo‐A^+^ oligodendrocytes was observed in the lesion core. Another distinction between lesion core and rim was the striking dissociation of both Nogo‐A^+^ oligodendrocytes and TPPP/p25^+^ oligodendrocyte numbers in the chronic stage IV. Nogo‐A^+^ oligodendrocytes and TPPP/p25^+^ oligodendrocyte numbers declined substantially at the lesion core while increasing at the rim.

## 4 DISCUSSION

Decades after the discovery of the neurite growth inhibitory protein, later referred to as Nogo‐A, the first generation of plasticity agonists is undergoing clinical testing [26, 44, 45] (NCT03935321; https://clinicaltrials.gov/). Plasticity agonists causally address the limited capacity of nerve fiber outgrowth and reconstitution of neurological function after SCI [1, 46, 47]. Here, we interrogated Nogo‐A expression after human SCI at regions of prevailing plasticity (“lesion rim,” formation of recovery‐related networks) or axonal damage and degeneration differentially (lesion core). Nogo‐A expression is dominated by TPPP/p25^+^ oligodendrocytes from acute to chronic SCI. Whereas Nogo‐A^+^/TPPP/p25^+^ oligodendrocytes increased over time after SCI in the perilesional rim reaching peak levels at chronic SCI stages, corresponding numbers at the lesion core only fluctuated. Here, after a transient and modest increase, a decline was observed between stages III and IV.

Nogo‐A expression was previously reported to be expressed by a variety of cells including motoneurons and oligodendrocytes [11, 43, 48]. Here, we report that just a limited number of Nogo‐A^+^ and TPPP/p25^+^ oligodendrocytes were present at the lesion core, an area of necrosis, subsequent neurodegeneration, and demyelination [29]. A possible explanation for persisting low numbers of Nogo‐A^+^ and TPPP/p25^+^ oligodendrocytes in the lesion core throughout stage IV might be that the adult glial scar formation and its specific extracellular matrix composition prevent oligodendrocyte precursors to be recruited to the lesion core even months and years after SCI [49].

A dynamic upregulation of Nogo‐A^+^ and TPPP/p25^+^ oligodendrocytes was detected specifically at the lesion rim, a crucial compartment for the formation and integration of recovery‐related networks (plasticity reservoir) [32]. Earlier rodent studies, however, described an overall stable, nonreactive Nogo‐A expression after SCI [10]. The substantial oligodendrogenesis reported after SCI implies a dynamic regulation and increase of Nogo‐A^+^ oligodendrocyte numbers [27]. The possibility that different antibodies used in animal models previously might have accounted for discrepancy of reported findings in human is unlikely as the applied Nogo‐A antibody detected the identical cellular and subcellular pattern in per se comparable control spinal cords as reported earlier [27, 28, 30, 33, 43].

Motoneurons are further known to express Nogo‐A [48]. Morphological alteration and a grossly damaged anterior horn in most cases precluded reliable quantification and analysis of Nogo‐A expression by motoneurons. However, as the main purpose of this study was to delineate localization and kinetics of plasticity restricting growth‐inhibiting molecule Nogo‐A after human SCI, we succeeded in finding that Nogo‐A^+^ oligodendrocytes comprised the most relevant cellular source of Nogo‐A [2, 12]. Our data suggest that the observed time‐dependent increase of Nogo‐A^+^ oligodendrocytes within the perilesional rim mirrors the dynamic activity of the perilesional re‐myelination and the endogenous attempt at repair in general. The putative relevance of an increasing number of Nogo‐A^+^ oligodendrocytes accounting for an increasing degree of plasticity inhibition preventing the formation of recovery‐related networks is further supported by clinical evidence since potential neurological recovery is confined to early stages after SCI [50, 51].

To further delineate the cellular substrate of Nogo‐A expression, we performed double labeling of Nogo‐A and TPPP/p25. We observed both Nogo‐A^+^ and Nogo‐A^−^ oligodendrocytes. Furthermore, we found coexpression of Nogo‐A^+^/TPPP/p25^+^ subpopulations of oligodendrocytes suggestive of a prevalent spectrum of oligodendrocytes in vicinity to the lesion area after SCI in humans. We did not observe a correlation of Nogo‐A expression on oligodendrocytes and axonal injury. The delayed increase of Nogo‐A expression appears not to be triggered by the axonal injury itself or only responds with some latency as APP^+^ profiles subsided with time after injury while Nogo‐A expression increased.

The study has limitations. Our cases only included formalin‐fixed paraffin‐embedded tissue, therefore additional analysis such as western blotting could not be performed, moreover quantification of proliferation was limited in postmortem tissue. Also, included cases represent a heterogeneous group of injury types, stages after SCI, and age. The study population, overall and in the respective groups, was small. However, reports on human tissue samples are scarce in literature as specimens are rarely available in neuropathological archives. Therefore, this study is in line with “best evidence available.”

We provide evidence for an astounding dynamic expression of the myelin‐associated growth inhibitor Nogo‐A in plasticity relevant regions after human SCI. The persistence of axon growth inhibitory protein Nogo‐A in plasticity relevant regions suggests a contribution to the maintenance of an environment that is hostile to neuroaxonal plasticity after human SCI. These data suggest a therapeutical window for anti‐Nogo‐A pathway associated treatments beyond 4 weeks including chronic SCI patients. Further studies will be necessary to investigate whether a combinational approach of biomaterials or scar‐degrading enzymes (e.g. ChABC) with an anti‐Nogo‐A therapy will be of additional benefit to overcome possible barriers of chronic SCI therapy.

## AUTHOR CONTRIBUTIONS

Carmen Schwaiger: Conception and design; acquisition of data; statistical analysis; execution; interpretation of data; critical review for important intellectual content. Thomas Haider: Conception and design; statistical analysis; interpretation of data; critical review for important intellectual content. Tobias Zrzavy: Acquisition of data; interpretation of data; critical review for important intellectual content. Verena Endmayr: Acquisition of data; execution; interpretation of data; critical review for important intellectual content. Victoria E. Gruber: Acquisition of data; execution; critical review for important intellectual content. Simon Hametner: Interpretation of data; critical review for important intellectual content. Gerda Ricken: Execution; critical review for important intellectual content. Jan M. Schwab: Conception and design; interpretation of data; critical review for important intellectual content. Romana Höftberger: Conception and design; acquisition of data; execution; interpretation of data; critical review for important intellectual content. All Authors have approved the final version of the manuscript.

## CONFLICTS OF INTEREST

The authors declare no conflicts of interest.

## Data Availability

All data can be made available from the corresponding author upon reasonable request and after approval from the ethics review board at the Medical University of Vienna, Vienna, Austria.
